# Supplementary material for: Compression-induced NF-κB activation sustains tumor cell survival in confinement by detoxifying aldehydes and promotes metastasis
Source: Nat Commun. 2025 Dec 14;17:778. doi: 10.1038/s41467-025-67452-7 (PMC12824153; doi:10.1038/s41467-025-67452-7)
Supplement: Supplementary file 1 — supplementary information [file 41467_2025_67452_MOESM1_ESM.pdf]

Supplementary Information

Files:

Supplementary Figs. 1-11

Title:

Compression-induced NF-κB activation sustains tumor cell survival in confinement by detoxifying aldehydes and promotes metastasis

Authors:

Bing Liu[1#], Min Liu[1#], Yajuan Zhang[2], Yifei Zhu[3], Dingpei Zhou[1], Hong Gao[1], Fan Yang[4], Dong Gao[1], Yun Zhao[1], Bangbao Tao[5 ✉], Feng Yao[2 ✉], Weiwei Yang[1,6,7 ✉]

Affiliations:

[1]Key Laboratory of Multi-Cell Systems, Shanghai Key Laboratory of Molecular Andrology, Center for Excellence in Molecular Cell Science, Chinese Academy of Sciences, Shanghai Institute of Biochemistry and Cell Biology, Shanghai, China.

[2]Department of Thoracic Surgery, Shanghai Chest Hospital, Shanghai Jiao Tong University, Shanghai, China.

[3]Department of Oncology, Fudan University Shanghai Cancer Center, Shanghai, China.

[4]Shenzhen Center for Disease Control and Prevention, Shenzhen, China.

[5]Department of Neurosurgery, XinHua Hospital School of Medicine, Shanghai Jiaotong University, Shanghai, China.

[6]Key Laboratory of Systems Health Science of Zhejiang Province, School of Life Science, Hangzhou Institute for Advanced Study, University of Chinese Academy of

Sciences, Hangzhou, China.

[7]Shanghai Academy of Natural Sciences (SANS), Shanghai, China.

[#]Equally Contributing Authors:

Bing Liu, Min Liu.

✉email:

Weiwei Yang, wyang@sibcb.ac.cn;

Feng Yao, yaofeng@shsmu.edu.cn;

Bangbao Tao, taobangbao@xinhuamed.com.cn;

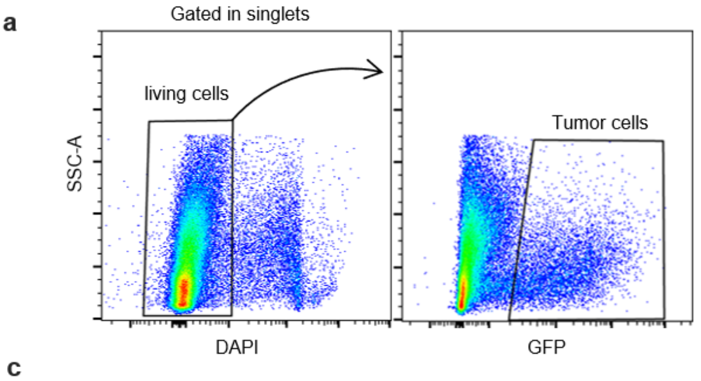

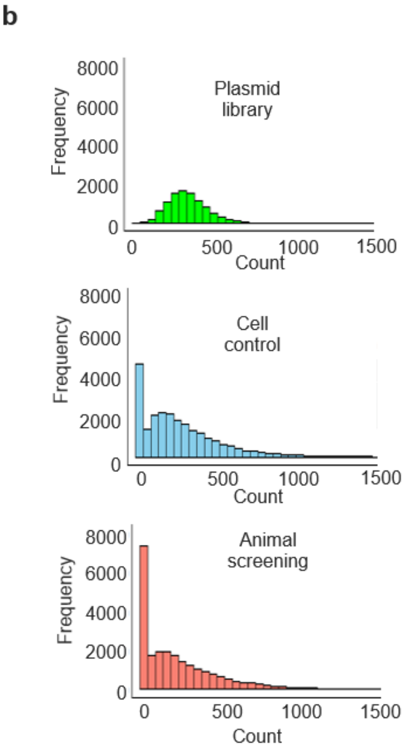

c

10 candidate genes: log_2(sgRNA fold change) ≤ -2, good sgRNA ≥ 5, *P*-value < 0.05

gene	log_2(sgRNA fold change)	good sgRNA	*p*-value
1.KYNU	-6.2491	5	0.018247
2.PAH	-6.0895	6	0.000545
3.PIK3C2A	-5.8893	5	0.026425
4.MTMR8	-5.8621	5	0.001912
5.PTS	-5.8077	5	0.029009
6.DGKI	-5.8072	5	0.019087
7.LHPP	-5.5244	5	0.03087
8.ALDH1B1	-5.5097	5	0.019985
9.RDH12	-5.4755	5	0.034092
10.MRI1	-5.4359	5	0.012626

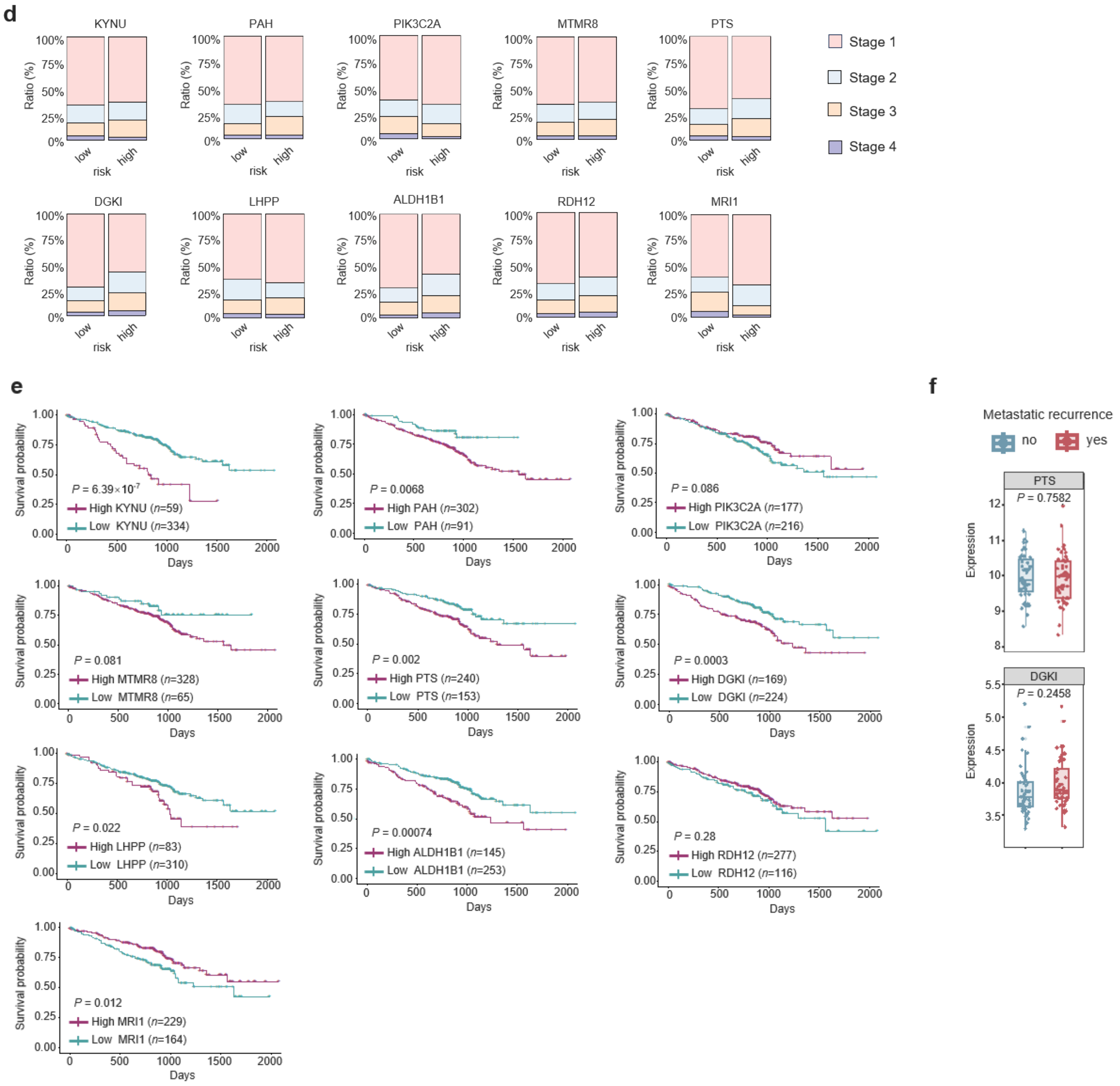

Supplementary Fig. 1 ALDH1B1 expression is associated with tumor metastasis

a Representative plots show the gating strategy for Live (left), GFP-positive A549 cells (right) from n = 9 mice. **b** Quality control of the CRISPR library showed > 95% sgRNA detection efficiency (upper). In the CRISPR screen, sgRNA depletion rates were 11.9% in control group (average reads/sgRNA = 273) (middle), compared to 19.5% depletion in the animal screening group (average reads/sgRNA = 231) (lower). **c** Ten candidate genes identified in screening that met the cut-off criteria (log_2 (sgRNA fold change) ≤ -2, good sgRNA count ≥ 5, $P < 0.05$), with ranking based on fold change. **d** Bar plots of proportional differences in different TMN stages between the ten candidate genes based on GEO dataset (GSE72094). **e** Overall survival analysis of ten candidate genes in human patients with lung adenocarcinoma based on GEO dataset (GSE72094). **f** Box plots show PTS and DGKI expression in lung cancer patients with (n = 49) and without (n = 47) metastatic recurrence based on GEO dataset (GSE37745). The center line represents the median, box limits the upper and lower quartiles, and whiskers the minimum and maximum values. P-values were calculated using two-tailed log-rank test (**e**) and two-tailed Student's t test (**f**). NS, not significant. Source data are provided as a Source Data file.

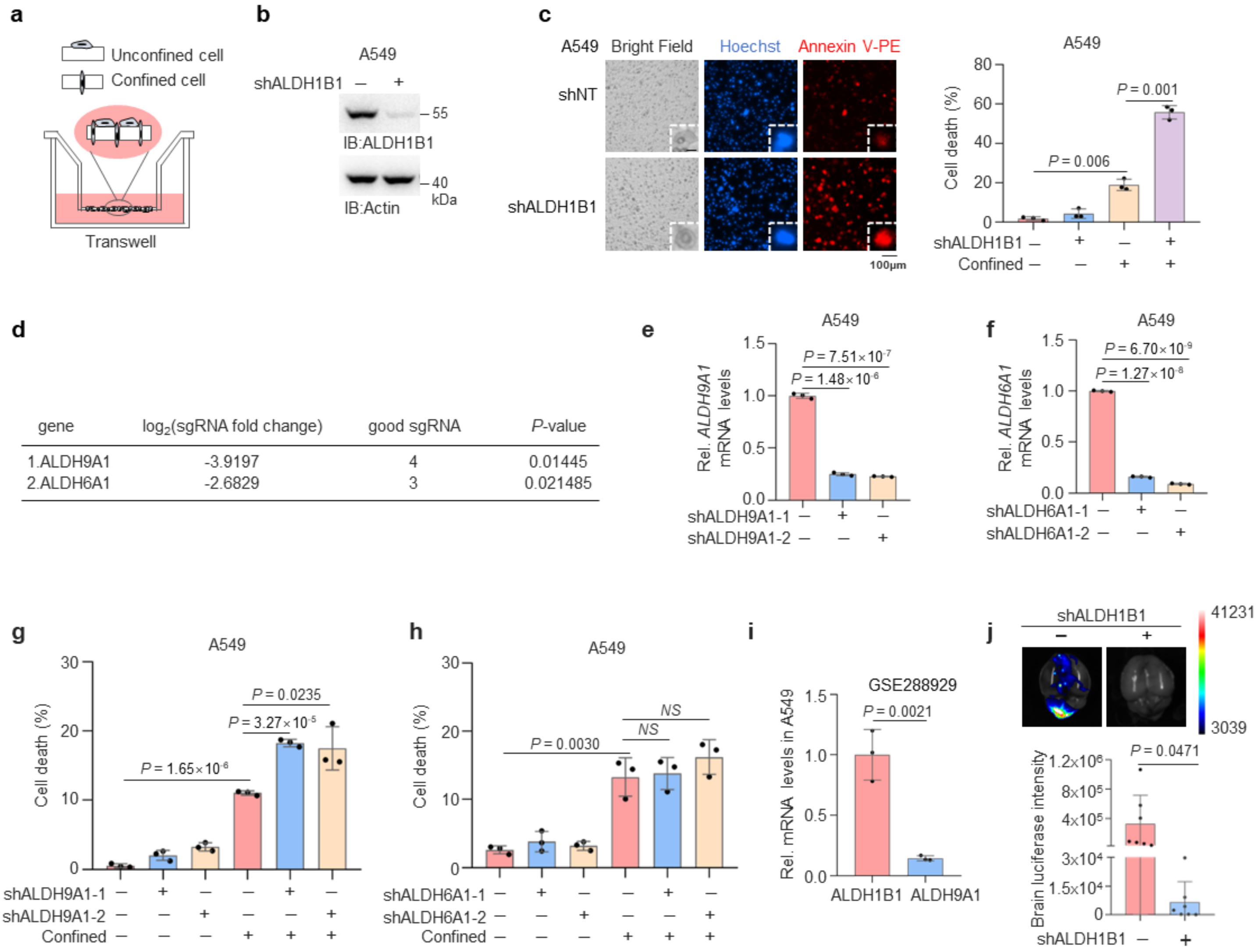

gene	$\log_2$(sgRNA fold change)	good sgRNA	*P*-value
1.ALDH9A1	-3.9197	4	0.01445
2.ALDH6A1	-2.6829	3	0.021485

Supplementary Fig. 2 ALDH1B1 promotes tumor cell survival in confining spaces

a Schematic models of confined and unconfined cells in transwell. **b** The protein levels of ALDH1B1 in A549 cells with or without ALDH1B1 depletion were detected by using immunoblotting analyses. **c** A549 cells with or without ALDH1B1 depletion were stained with Annexin-V-PE and photographed in situ after 6 hours of transwell migration. Representative images of the dead confined cells from n = 3 biologically independent experiments were presented (left), with quantified percentages of unconfined and confined cell death (right). Scale bar represents 15 μm (zoomed-in images). **d** The two candidate ALDH isoform genes identified in the screening that met the cut-off criteria ($\log_2$(sgRNA fold change) ≤ -1, P < 0.05), with ranking based on fold change.**e** *ALDH9A1* mRNA levels in A549 cells with or without ALDH9A1 depletion were quantified by qPCR. **f** *ALDH6A1* mRNA levels in A549 cells with or without ALDH6A1 depletion were quantified by qPCR. **g, h** A549 cells with or without ALDH9A1

depletion were stained with Annexin V-PE and imaged in situ after 6 hours transwell migration. The percentages of unconfined and confined cell death were quantified (**g**). A549 cells with or without ALDH6A1 depletion were similarly processed and analyzed (**h**). **i** Relative expression of ALDH1B1 and ALDH9A1 in A549 cells was analyzed using GEO dataset GSE288929. **j** Representative images of brain metastasis (Fig. 1**h)** from *n* = 7 mice per group are shown (upper). Data are presented as mean ± SD of luciferase intensities per mouse (lower). Data are presented as mean ± SD (*n* = 3 biologically independent experiments) (**c**, **e**-**i**). Immunoblotting experiments were performed with the indicated antibodies. Data are representative of three independent experiments (**b**). *P*-values were calculated using unpaired two-tailed Student's *t* test (**c**, **e**-**i**). Rel., relative. Source data are provided as a Source Data file.

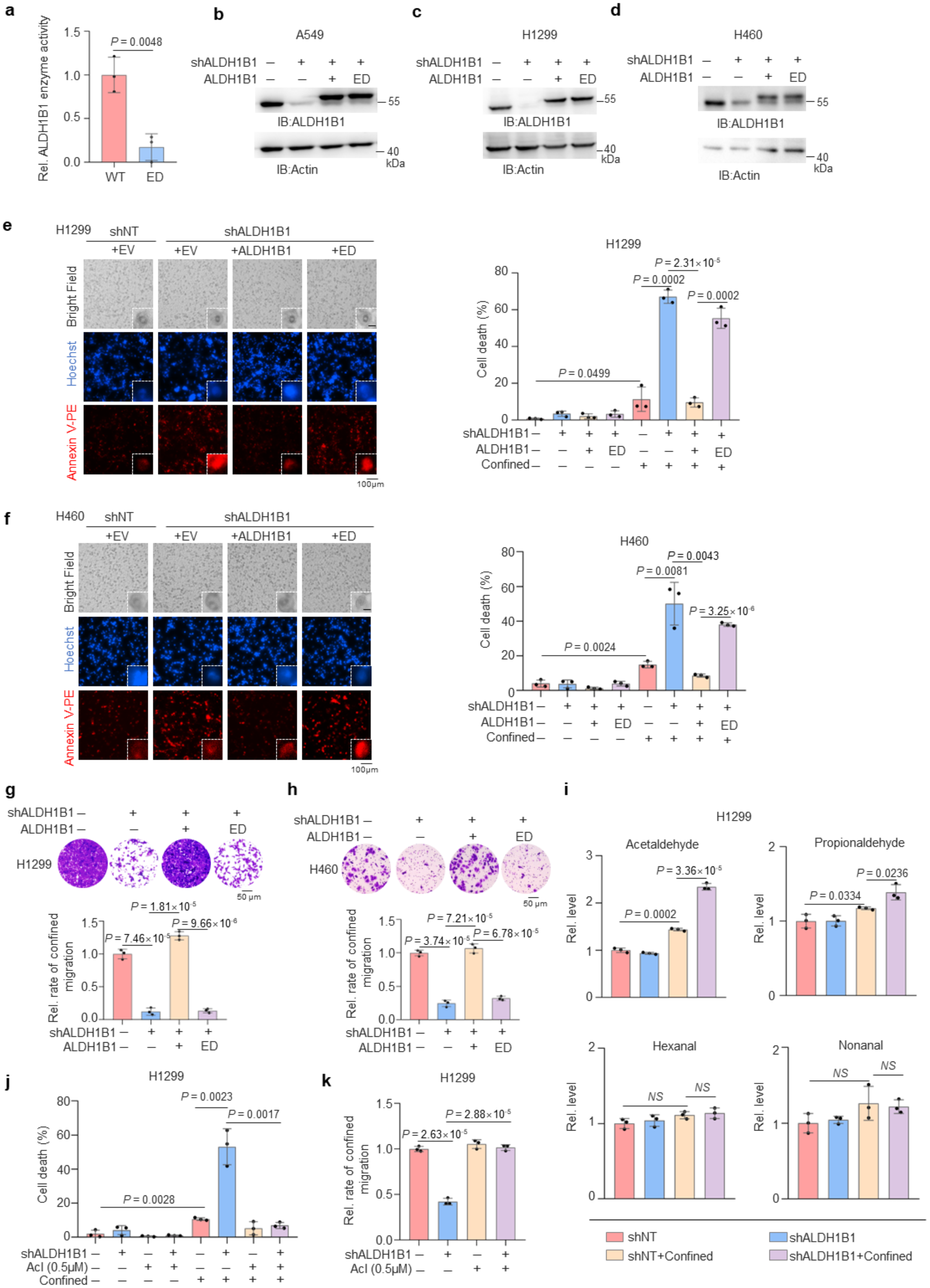
a
Rel. ALDH1B1 enzyme activity
P = 0.0048
1.5
1.0
0.5
0.0
WT
ED
b
A549
shALDH1B1 – + + +
ALDH1B1 – – + ED
55
IB:ALDH1B1
40
kDa
IB:Actin
c
H1299
shALDH1B1 – + + +
ALDH1B1 – – + ED
IB:ALDH1B1
IB:Actin
d
H460
shALDH1B1 – + + +
ALDH1B1 – – + ED
IB:ALDH1B1
IB:Actin
e
H1299
shNT
shALDH1B1
+EV
+ALDH1B1
+ED
Bright Field
Hoechst
Annexin V-PE
100μm
Cell death (%)
P = 0.0499
P = 0.0002
P = 2.31×10-5
P = 0.0002
shALDH1B1 – + + + – + + +
ALDH1B1 – – + ED – – + ED
Confined – – – – + + + +
f
H460
P = 0.0024
P = 0.0081
P = 0.0043
P = 3.25×10-6
g
shALDH1B1 – + + +
ALDH1B1 – – + ED
H1299
50 μm
Rel. rate of confined migration
P = 7.46×10-5
P = 1.81×10-5
P = 9.66×10-6
h
H460
P = 3.74×10-5
P = 7.21×10-5
P = 6.78×10-5
i
H1299
Acetaldehyde
Propionaldehyde
Hexanal
Nonanal
Rel. level
P = 0.0002
P = 3.36×10-5
P = 0.0334
P = 0.0236
NS
shNT
shNT+Confined
shALDH1B1
shALDH1B1+Confined
j
H1299
P = 0.0028
P = 0.0023
P = 0.0017
shALDH1B1 – + – + – + – +
AcI (0.5μM) – – + + – – + +
Confined – – – – + + + +
k
H1299
P = 2.63×10-5
P = 2.88×10-5
shALDH1B1 – + – +
AcI (0.5μM) – – + +

Supplementary Fig. 3 ALDH1B1 activity is essential for tumor cell survival and migration in confining spaces

a Flag-ALDH1B1(WT) or Flag-ALDH1B1 E285A (ED) were immunoprecipitated from A549 cells to determine ALDH1B1 enzymatic activity. **b-d** ALDH1B1-depleted A549, H1299 or H460 cells were reconstituted with Flag-ALDH1B1 or Flag-ALDH1B1 ED, ALDH1B1 expression was examined by immunoblotting analyses. **e-h** ALDH1B1-depleted H1299 cells or H460 cells were reconstituted with ALDH1B1 WT or ED. The cells were stained with Annexin-V-PE and photographed in situ after 6 hours of transwell migration. Representative images of the dead confined cells from n = 3 biologically independent experiments were presented (**e**, left; **f**, left), with quantified percentages of unconfined and confined cell death (**e**, right; **f**, right). Scale bar represents 15 μm (zoomed-in images). Transwell migration assays were performed after 18 hours of culture. Representative images from n = 3 biologically independent experiments (**g**, upper; **h**, upper) and statistical analyses (**g**, lower; **h**, lower) of the migrated cells are shown. **i** H1299 cells expressing shNT or shALDH1B1were performed transwell migration assays. Collecting cells with or without confinement for aldehyde assays. **j, k** ALDH1B1-depleted H1299 cells were treat with or without 0.5 μM aldehyde scavenger Acloproxalap. The percentages of unconfined and confined cell death were quantified (**j**). Transwell migration assays were performed and statistical analyses of the migrated cells are shown (**k**). Data are presented as mean ± SD (n = 3 biologically independent experiments) (**a**, **e-k**). Immunoblotting experiments were performed with the indicated antibodies. Data are representative of three independent experiments (**b-d**). *P*-values were calculated using unpaired two-tailed Student's t test (**a**, **e-k**). NS, not significant. Rel., relative. Source data are provided as a Source Data file.

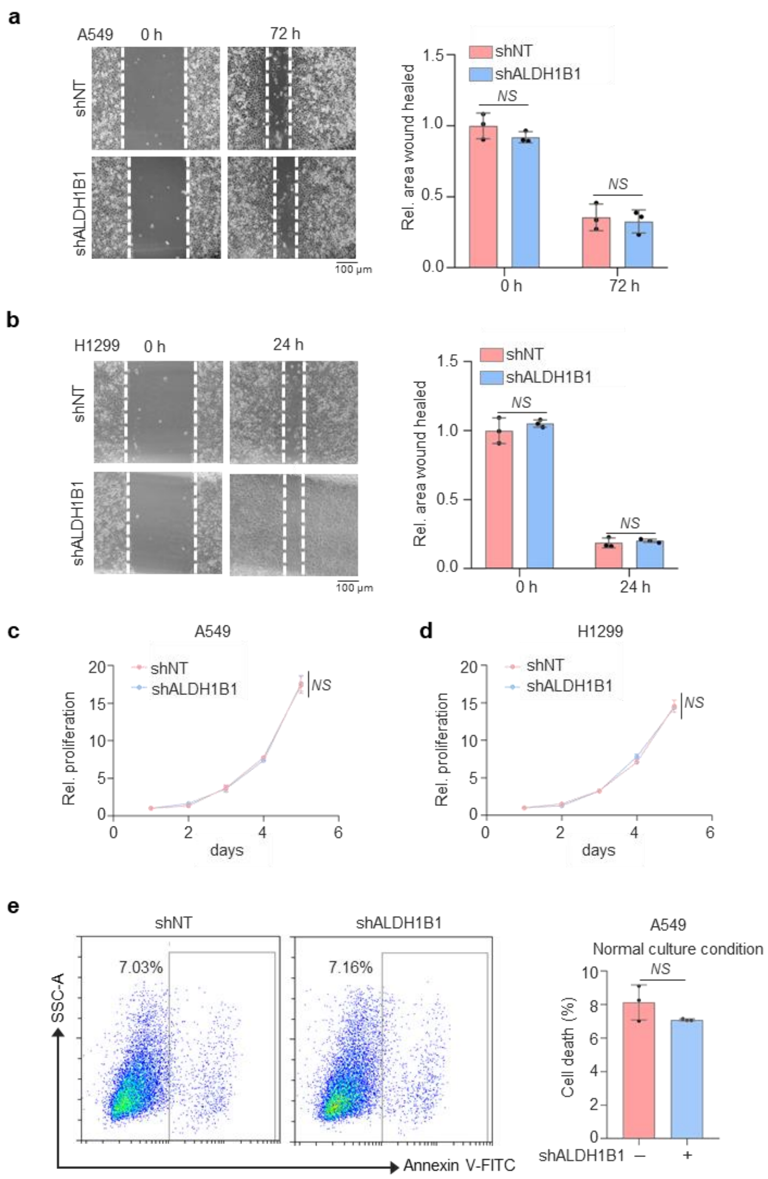

Supplementary Fig. 4 ALDH1B1 depletion does not influence the mobility, proliferation and survival of the cells without confinement

a, b A549 or H1299 cells with or without ALDH1B1 depletion were performed wound healing assay. Representative images of wound width changes from *n* = 3 biologically independent experiments were presented (**a**, left; **b**, left). Relative changes of wound width were normalized to those cells without ALDH1B1 depletion (**a**, right; **b**, right). **c, d** A549 or H1299 cells with or without ALDH1B1 depletion were performed cell proliferation assay. Relative proliferation of cells with or without ALDH1B1 depletion was normalized to day 1 respectively (**c**, *n* = 5 biologically independent experiments; **d**, *n* = 6 biologically independent experiments). **e** Cell death was assessed by flow cytometry in A549 cells with or without ALDH1B1 depletion after Annexin-V-FITC staining. A representative flow cytometry plot from *n* = 3 biologically independent experiments shows the gating strategy for dead cells (left) and

the corresponding quantified percentages of cell death (right). Data are presented as mean ± SD. *P*-values were calculated using unpaired two-tailed Student's t test (**a**-**e**). NS, not significant. Rel., relative. Source data are provided as a Source Data file.

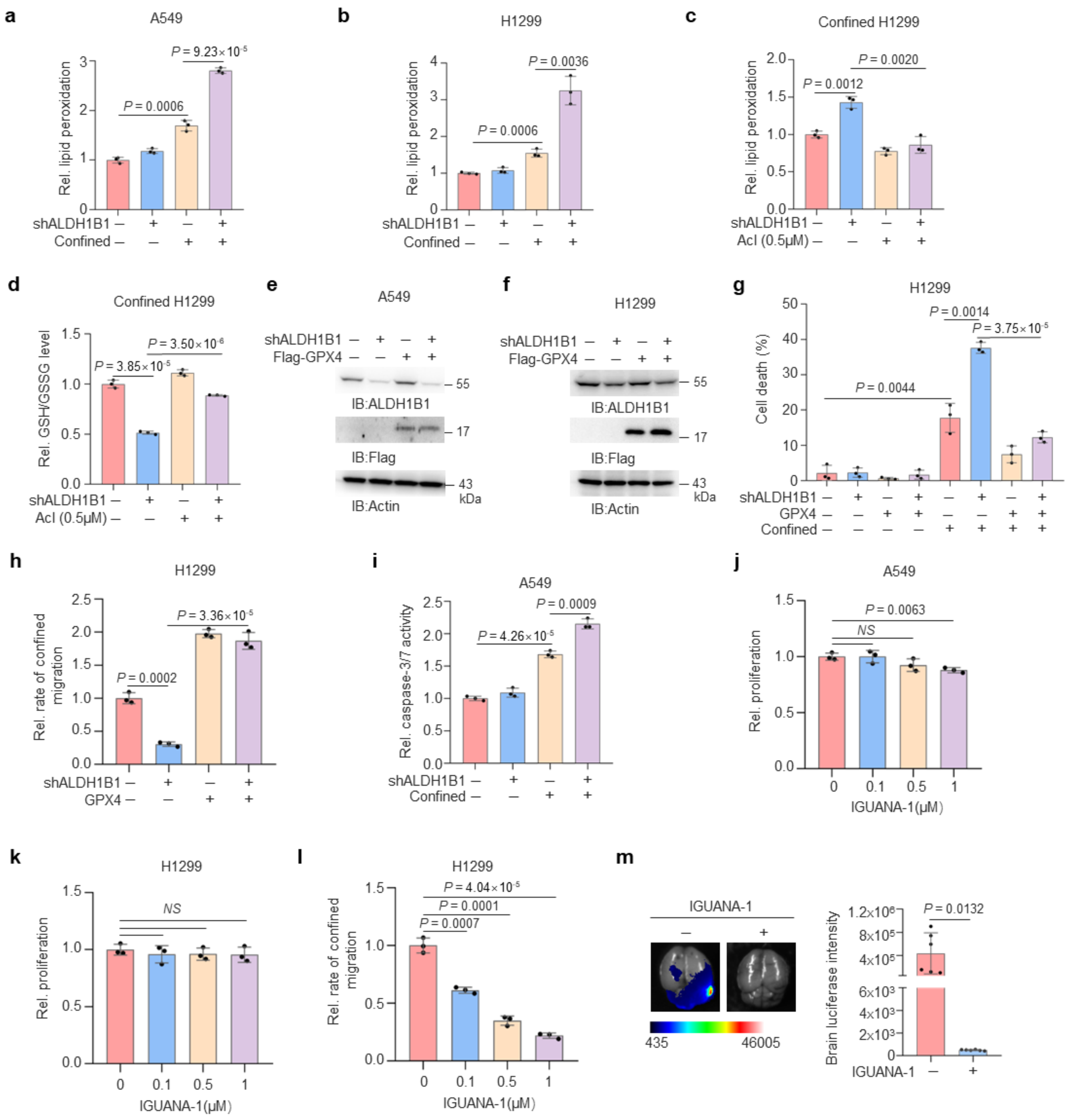

Supplementary Fig. 5 ALDH1B1 inhibits ferroptosis and promotes distant metastasis

a, b A549 or H1299 cells with or without ALDH1B1 depletion were stained with BODIPY 581/591 C11 and photographed in situ after 6 hours of transwell migration. Lipid peroxidation in unconfined and confined cells was assessed. **c, d** ALDH1B1-depleted H1299 cells were treat with or without 0.5 µM aldehyde scavenger Acloproxalap. The cells were stained with BODIPY 581/591 C11 and photographed in situ after 6 hours of transwell migration. Lipid peroxidation in confined cells was assessed (**c**). Measurement of GSH and GSSG levels in these confined cells. The GSH/GSSG ratio are shown (**d**).

e, f ALDH1B1-depleted A549 and H1299 cells were overexpressed with Flag-EV or Flag-GPX4. Immunoblotting analyses were performed. **g, h** ALDH1B1-depleted H1299 cells were overexpressed with EV or GPX4. The percentages of unconfined and confined cell death were quantified (**g**). Transwell migration assays were performed and statistical analyses of the migrated cells are shown (**h**). **i** A549 cells expressing shNT or shALDH1B1 were performed transwell migration assays. Collecting cells with or without confinement for caspase-3/7 activity assays. **j, k** A549 and H1299 cells were treated with 0/0.1/0.5/1 μM IGUANA-1. Cell proliferation assay was performed for 24 hours. Relative cell proliferation of A549 and H1299 cells was normalized to 0μM respectively. **l** H1299 cells were treated with 0/0.1/0.5/1 μM IGUANA-1. Transwell migration assays were performed and statistical analyses of the migrated cells are shown. **m** Representative images of brain metastasis (Fig. 2**l**) from $n = 6$ mice per group are shown (left). Data represent mean ± SD of luciferase intensities per mouse (right). Data are presented as mean ± SD ($n = 3$ biologically independent experiments) (**a-d**, **g-l**). Immunoblotting experiments were performed with the indicated antibodies. Data are representative of three independent experiments (**e**, **f**). *P*-values were calculated using unpaired two-tailed Student's t test (**a-d**, **g-m**). NS, not significant. Rel., relative. Source data are provided as a Source Data file.

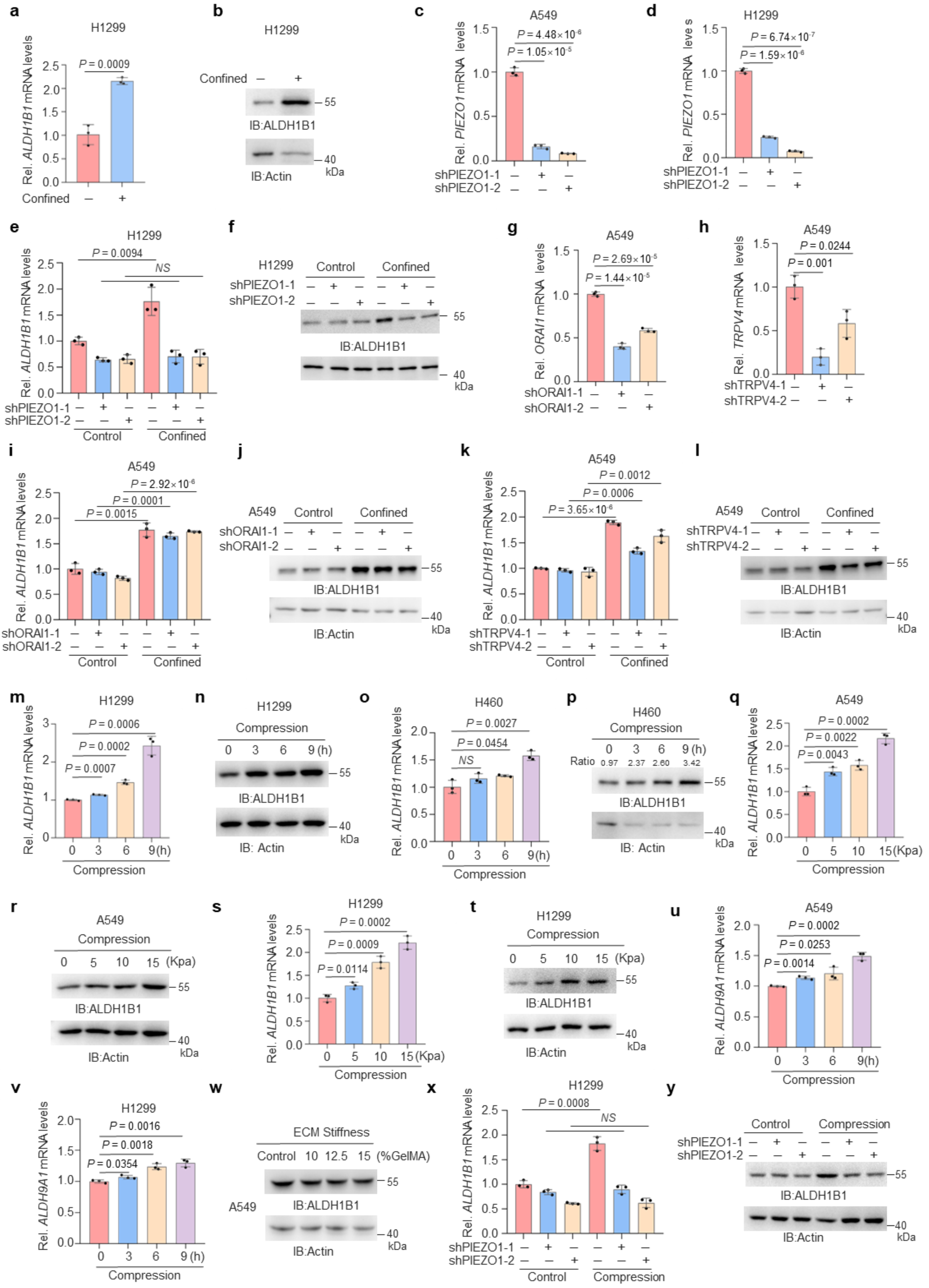

a
H1299
P = 0.0009
Rel. ALDH1B1 mRNA levels
Confined – +
b
H1299
Confined – +
IB:ALDH1B1
IB:Actin
55
40
kDa
c
A549
P = 4.48×10-6
P = 1.05×10-5
Rel. PIEZO1 mRNA levels
shPIEZO1-1 – + –
shPIEZO1-2 – – +
d
H1299
P = 6.74×10-7
P = 1.59×10-6
Rel. PIEZO1 mRNA levels
shPIEZO1-1 – + –
shPIEZO1-2 – – +
e
H1299
P = 0.0094
NS
Rel. ALDH1B1 mRNA levels
shPIEZO1-1
shPIEZO1-2
Control
Confined
f
H1299
Control
Confined
shPIEZO1-1
shPIEZO1-2
IB:ALDH1B1
55
40
kDa
g
A549
P = 2.69×10-5
P = 1.44×10-5
Rel. ORAI1 mRNA levels
shORAI1-1 – + –
shORAI1-2 – – +
h
A549
P = 0.0244
P = 0.001
Rel. TRPV4 mRNA levels
shTRPV4-1 – + –
shTRPV4-2 – – +
i
A549
P = 2.92×10-6
P = 0.0001
P = 0.0015
Rel. ALDH1B1 mRNA levels
shORAI1-1
shORAI1-2
Control
Confined
j
A549
Control
Confined
shORAI1-1
shORAI1-2
IB:ALDH1B1
IB:Actin
55
40
kDa
k
A549
P = 0.0012
P = 0.0006
P = 3.65×10-6
Rel. ALDH1B1 mRNA levels
shTRPV4-1
shTRPV4-2
Control
Confined
l
A549
Control
Confined
shTRPV4-1
shTRPV4-2
IB:ALDH1B1
IB:Actin
55
40
kDa
m
H1299
P = 0.0006
P = 0.0002
P = 0.0007
Rel. ALDH1B1 mRNA levels
0 3 6 9(h)
Compression
n
H1299
Compression
0 3 6 9 (h)
IB:ALDH1B1
IB: Actin
55
40
kDa
o
H460
P = 0.0027
P = 0.0454
NS
Rel. ALDH1B1 mRNA levels
0 3 6 9(h)
Compression
p
H460
Compression
0 3 6 9 (h)
Ratio 0.97 2.37 2.60 3.42
IB:ALDH1B1
IB: Actin
55
40
kDa
q
A549
P = 0.0002
P = 0.0022
P = 0.0043
Rel. ALDH1B1 mRNA levels
0 5 10 15(Kpa)
Compression
r
A549
Compression
0 5 10 15 (Kpa)
IB:ALDH1B1
IB:Actin
55
40
kDa
s
H1299
P = 0.0002
P = 0.0009
P = 0.0114
Rel. ALDH1B1 mRNA levels
0 5 10 15(Kpa)
Compression
t
H1299
Compression
0 5 10 15 (Kpa)
IB:ALDH1B1
IB:Actin
55
40
kDa
u
A549
P = 0.0002
P = 0.0253
P = 0.0014
Rel. ALDH9A1 mRNA levels
0 3 6 9(h)
Compression
v
H1299
P = 0.0016
P = 0.0018
P = 0.0354
Rel. ALDH9A1 mRNA levels
0 3 6 9 (h)
Compression
w
ECM Stiffness
Control 10 12.5 15 (%GelMA)
A549
IB:ALDH1B1
IB:Actin
55
40
kDa
x
H1299
P = 0.0008
NS
Rel. ALDH1B1 mRNA levels
shPIEZO1-1
shPIEZO1-2
Control
Compression
y
Control
Compression
shPIEZO1-1
shPIEZO1-2
IB:ALDH1B1
IB:Actin
55
40
kDa

Supplementary Fig. 6 ALDH1B1 is upregulated upon compression

a, b *ALDH1B1* mRNA (**a**) and protein (**b**) levels in H1299 cells cultured under confinement or unconfinement conditions were analyzed. **c, d** *PIEZO1* mRNA levels were measured in A549 and H1299 cells with or without PIEZO1 depletion. **e, f** *ALDH1B1* mRNA (**e**) and protein (**f**) levels were analyzed in H1299 cells with or without PIEZO1 depletion under confinement or unconfinement conditions. **g,h** *ORAI1* (**g**) and *TRPV4* (**h**) mRNA levels in A549 cells with or without respective gene depletion were quantified. **i, j** *ALDH1B1* mRNA (**i**) and protein (**j**) levels in A549 cells with or without ORAI1 depletion under confinement or unconfinement conditions were analyzed. **k, l** *ALDH1B1* mRNA (**k**) and protein (**l**) levels in A549 cells with or without TRPV4 depletion under confinement or unconfinement conditions were analyzed. **m-p** H1299 or H460 cells were treated with or without compression (5 kPa) for various time points. *ALDH1B1* mRNA (**m**, **o**) and protein (**n**, **p**) levels were analyzed. ALDH1B1 band intensities were normalized to β-actin (**p**). **q-t** A549 or H1299 cells were treated with or without compression (5, 10 or 15 kPa) for 6 hours. *ALDH1B1* mRNA (**q**, **s**) and protein (**r**, **t**) levels were analyzed. **u, v** *ALDH9A1* mRNA levels in A549 or H1299 cells treated with or without compression (5 kPa) for 6 hours were measured. **w** ALDH1B1 protein levels in A549 cells cultured under stiff or non-stiff conditions for 12 hours were examined. **x, y** H1299 cells, with or without PIEZO1 depletion, were treated with or without compression (5 kPa) for 6 hours. *ALDH1B1* mRNA (**x**) and protein (**y**) levels were analyzed. Data are presented as mean ± SD (*n* = 3 biologically independent experiments) (**a**, **c-e**, **g-i**, **k**, **m, o, q, s, u, v, x**). Immunoblotting experiments were performed with the indicated antibodies. Data are representative of three independent experiments (**b**, **f, j, l, n, p, r, t, w, y**). *P*-values were calculated using unpaired two-tailed Student's *t* test (**a**, **c-e**, **g-i**, **k**, **m, o, q, s, u, v, x**). NS, not significant. Rel., relative. Source data are provided as a Source Data file.

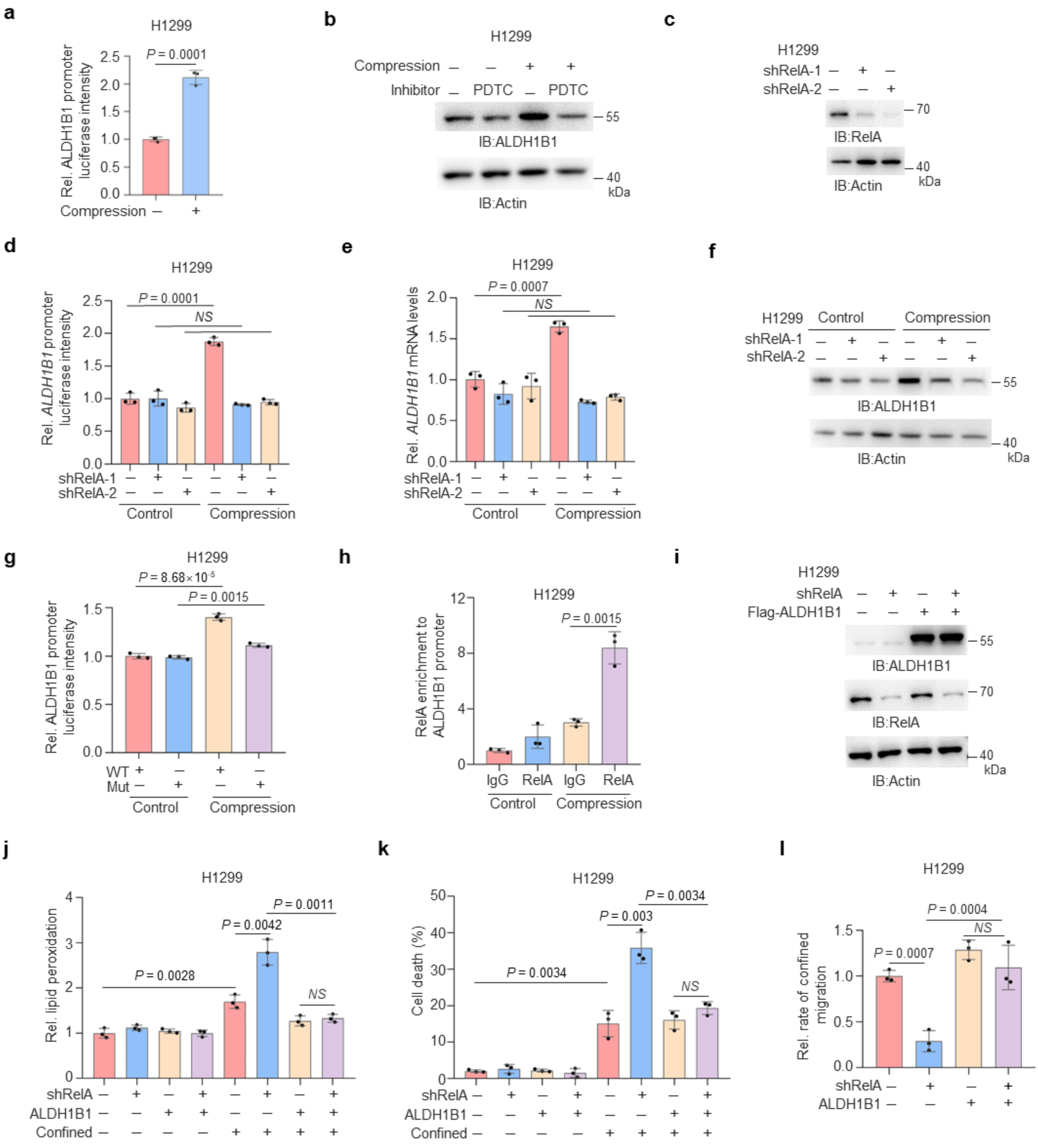

Supplementary Fig. 7 NF-kB regulates ALDH1B1 expression in H1299 cells

a H1299 cells transfected with the luciferase reporter containing the ALDH1B1 promoter. The transfected cells were treated with or without compression (5 kPa) for 6 hours. Relative luciferase activity was normalized to controls. **b** H1299 cells treated with or without the inhibitor of NFκB (PDTC, 5 μM) subjected to compression (5 kPa) for 6 hours. ALDH1B1 protein levels were assessed. **c** RelA protein levels in H1299 cells with or without RelA depletion were detected. **d** H1299 cells with or

without RelA depletion, transfected with the ALDH1B1 promoter-reporter, were treated with or without compression for 6 hours. Relative luciferase activity was normalized to controls. **e, f** H1299 cells with or without RelA depletion treated with or without compression (5 kPa) for 6 hours. *ALDH1B1* mRNA levels were analyzed (**e**). ALDH1B1 protein levels were detected (**f**). **g** H1299 cells transfected with ALDH1B1 promoter-reporter (WT or mutant). were treated with or without compression (5 kPa) for 6 hours. Relative luciferase activity was normalized to controls. **h** H1299 cells treated with or without compression for 6 hours were subjected to ChIP assays using indicated antibodies and primers targeting binding sites. Relative DNA levels were normalized to input and IgG controls. **i** RelA-depleted A549 cells overexpressing EV or ALDH1B1 were analyzed. The samples derive from the same experiment but different gels for ALDH1B1 and another for RelA, Actin were processed in parallel. **j-l** RelA-depleted H1299 cells overexpressing EV or ALDH1B1 were analyzed after transwell migration. Lipid peroxidation was assessed by BODIPY 581/591 C11 staining (**j**). Cell death percentages were quantified by Annexin V-PE staining (**k**). Migrated cells were quantified (**l**). Data are presented as mean ± SD (n = 3 biologically independent experiments) (**a**, **d**, **e**, **g**, **h**, **j-l**). Immunoblotting experiments were performed with the indicated antibodies. Data are representative of three independent experiments (**b, c, f, i**). *P*-values were calculated using unpaired two-tailed Student's t test (**a**, **d**, **e**, **g**, **h**, **j-l**). NS, not significant. Rel., relative. Source data are provided as a Source Data file.

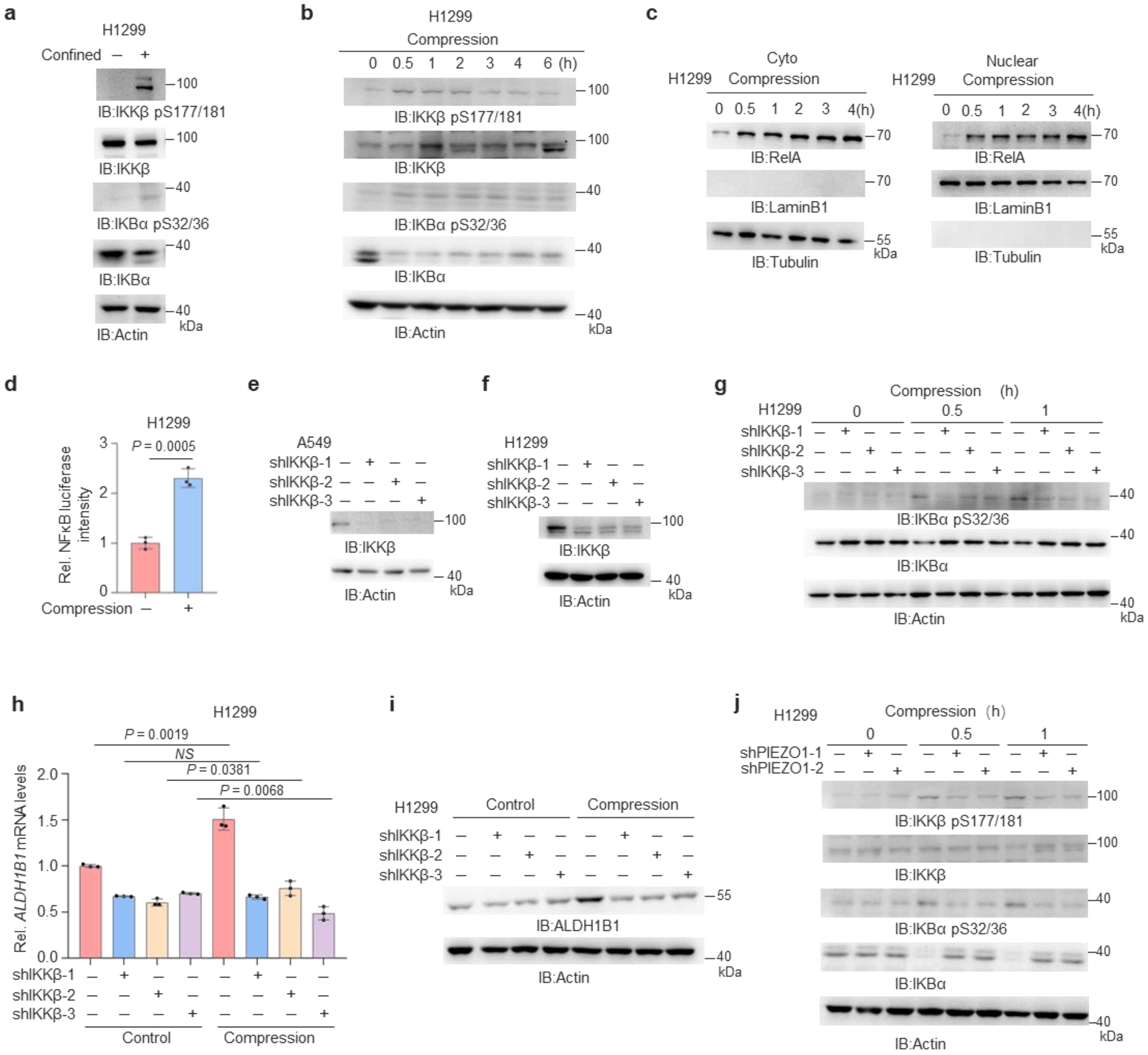

Supplementary Fig. 8 Piezo1 activates NF-kB signaling to upregulate ALDH1B1 in H1299 cells

a H1299 cells were treated with or without confinement and analyzed by immunoblotting. The samples derive from the same experiment but different gels for IKKβ, IKBα pS32/36, another for IKKβ pS177/181, another for IKBα and another for Actin were processed in parallel. **b** H1299 cells were treated with or without compression (5 kPa) at different time and analyzed by immunoblotting. The samples derive from the same experiment but different gels for IKKβ pS177/181, IKBα pS32/36 and another for IKKβ, IKBα, Actin were processed in parallel. **c** H1299 cells were treated with or without compression (5 kPa) at different time. The cytosolic and nuclear fractions were prepared and analyzed by immunoblotting. The samples derive from the same experiment but different gels for RelA and

another for LaminB1, Tubulin were processed in parallel. **d** H1299 cells were transfected with NF-κB-Luc reporter treated with or without compression (5 kPa). The relative luciferase activities were normalized to those of the cells without compression. **e, f** The protein levels of IKKβ in A549 and H1299 cells with or without IKKβ depletion were detected by using immunoblotting analysis. **g** H1299 cells with or without IKKβ depletion were treated with or without compression (5 kPa). Immunoblotting analyses were performed. The samples derive from the same experiment but different gels for IKBα pS32/36, and another for IKBα, Actin were processed in parallel. **h, i** H1299 cells, with or without IKKβ depletion, were treated with or without compression (5 kPa). *ALDH1B1* mRNA levels were quantified by qPCR (**h**), and protein levels of ALDH1B1 were analyzed by immunoblotting (**i**). **j** Immunoblotting analysis of H1299 cells, with or without PIEZO1 depletion, treated with or without compression (5 kPa). The samples derive from the same experiment but different gels for IKKβ pS177/181, IKBα pS32/36, another for IKKβ, Actin and another for IKBα were processed in parallel.Data are presented as mean ± SD (n = 3 biologically independent experiments) (**d**, **h**). Immunoblotting experiments were performed with the indicated antibodies. Data are representative of three independent experiments (**a- c, e-g, i, j**). P-values were calculated using unpaired two-tailed Student's t test (**d**, **h**). NS, not significant. Rel., relative. Source data are provided as a Source Data file.

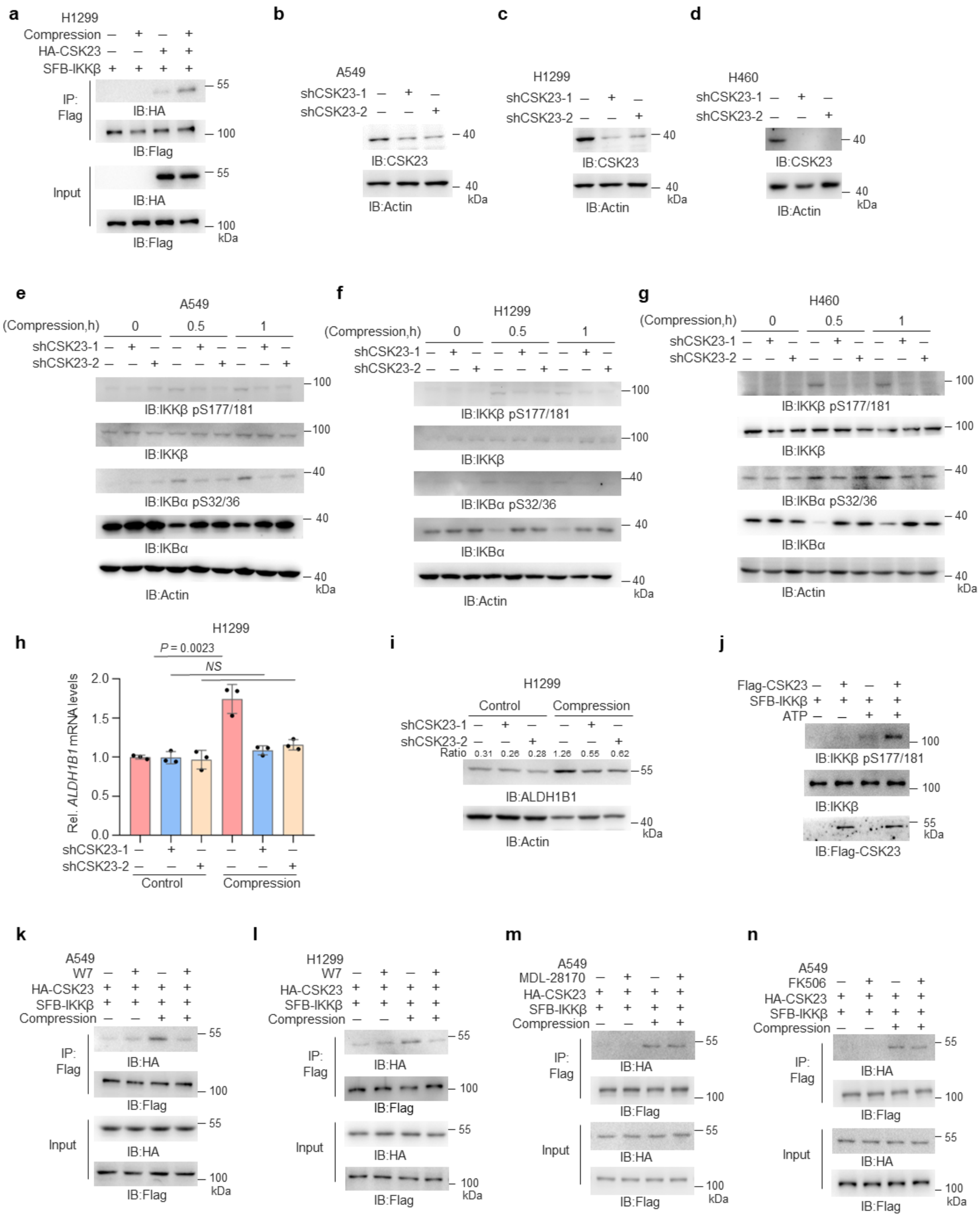

Supplementary Fig. 9 CSK23 is required for IKKβ activation and ALDH1B1 expression in confined cells

a, H1299 cells co-transfected with HA-CSK23 and SFB-IKKβ were treated with or without compression (5 kPa) for 2 hours and a co-immunoprecipitation (co-IP) assay was performed. **b-d** The

protein levels of CSK23 in A549, H1299 or H460 cells with or without CSK23 depletion were detected by using immunoblotting analysis. **e-g** A549, H1299 or H460 cells with or without CSK23 depletion were treated with or without compression (5 kPa) for different time points. Immunoblotting analyses were performed. The samples derive from the same experiment but different gels for IKKβ pS177/181, IKBα pS32/36 and another for IKKβ, IKBα and another for Actin were processed in parallel (**e**). The samples derive from the same experiment but different gels for IKKβ pS177/181, another for IKKβ, Actin, another for IKBα pS32/36 and another for IKBα were processed in parallel (**f**). The samples derive from the same experiment but different gels for IKKβ pS177/181, IKBα pS32/36, Actin and another for IKKβ, IKBα were processed in parallel (**g**). **h, i** H1299 cells with or without CSK23 depletion were treated with or without compression (5 kPa) for 6 hours. *ALDH1B1* mRNA levels were quantified by qPCR (**h**). ALDH1B1 protein levels were detected by immunoblotting analysis, and ALDH1B1 band intensities were normalized to β-actin (**i**). **j** *In vitro* kinase assay was performed by combining precipitated SFB-IKKβ and FLAG-CSK23 from A549 cells. The samples derive from the same experiment but different gels for IKKβ pS177/181 and another for IKKβ, CSK23 were processed in parallel. **k, l** A549 or H1299 cells co-transfected with HA-CSK23 and SFB-IKKβ were treated with or without the inhibitor of CaM (W7, 5 μM). These cells were then treated with or without compression (5 kPa) for 2 hours and a co-IP assay was performed. **m, n** A549 co-transfected with HA-CSK23 and SFB-IKKβ with or without the inhibitor of calpain (MDL-28170, 5 μM) and calcineurin (FK506, 5 μM). These cells were then treated with or without mechanical compression (5 kPa) for 2 hours, and a co-IP analysis were performed. Data are presented as mean ± SD ($n = 3$ biologically independent experiments) (**h**). Immunoblotting experiments were performed with the indicated antibodies. Data are representative of three independent experiments (**a-g, i-n**). *P*-values were calculated using unpaired two-tailed Student's *t* test (**h**). NS, not significant. Rel., relative. Source data are provided as a Source Data file.

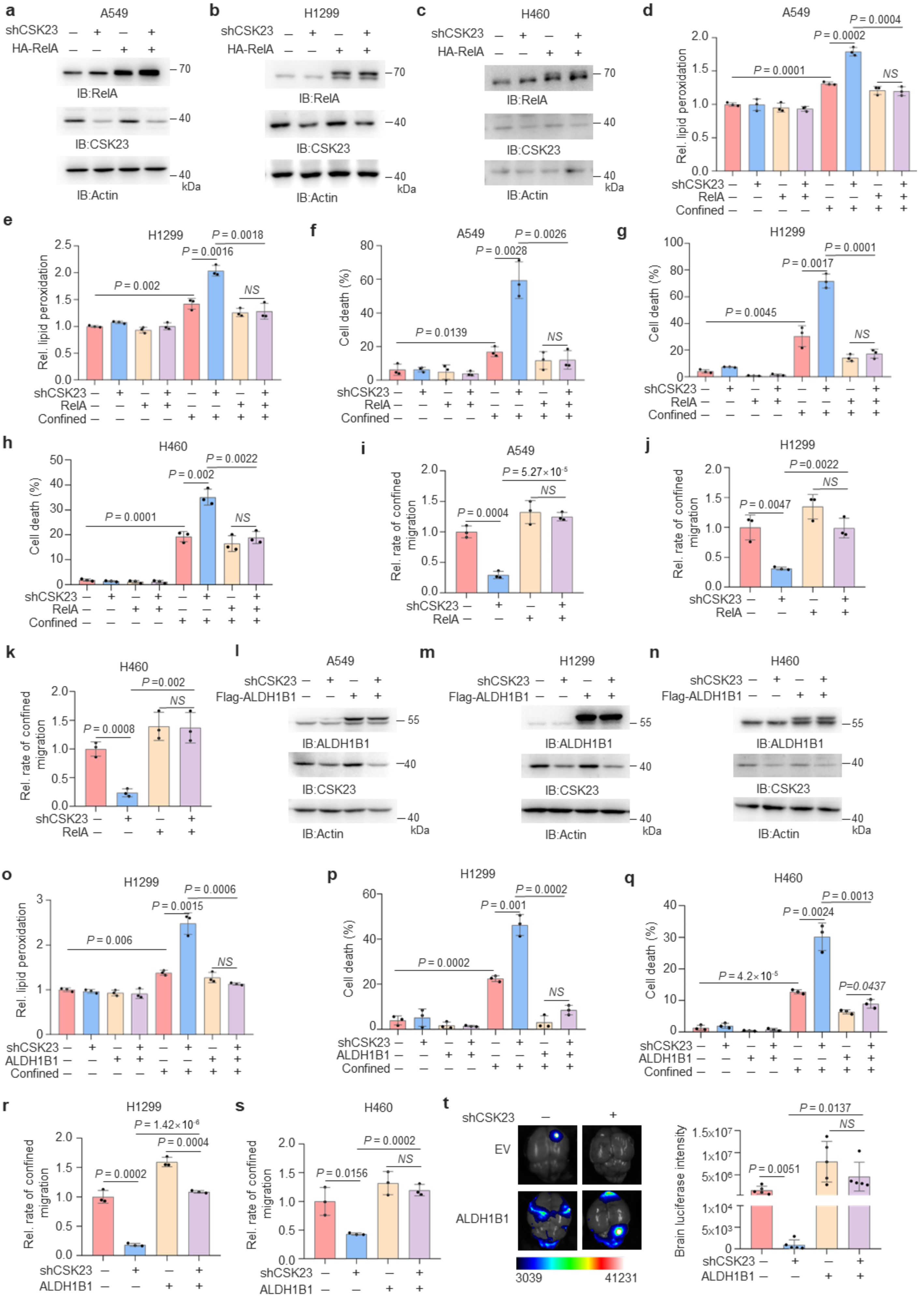
a
A549
shCSK23 – + – +
HA-RelA – – + +
IB:RelA
IB:CSK23
IB:Actin
70
40
40
kDa
b
H1299
shCSK23 – + – +
HA-RelA – – + +
IB:RelA
IB:CSK23
IB:Actin
70
40
40
kDa
c
H460
shCSK23 – + – +
HA-RelA – – + +
IB:RelA
IB:CSK23
IB:Actin
70
40
40
kDa
d
A549
Rel. lipid peroxidation
P = 0.0001
P = 0.0002
P = 0.0004
NS
shCSK23 – + – + – + – +
RelA – – + + – – + +
Confined – – – – + + + +
e
H1299
Rel. lipid peroxidation
P = 0.002
P = 0.0016
P = 0.0018
NS
shCSK23 – + – + – + – +
RelA – – + + – – + +
Confined – – – – + + + +
f
A549
Cell death (%)
P = 0.0139
P = 0.0028
P = 0.0026
NS
shCSK23 – + – + – + – +
RelA – – + + – – + +
Confined – – – – + + + +
g
H1299
Cell death (%)
P = 0.0045
P = 0.0017
P = 0.0001
NS
shCSK23 – + – + – + – +
RelA – – + + – – + +
Confined – – – – + + + +
h
H460
Cell death (%)
P = 0.0001
P = 0.002
P = 0.0022
NS
shCSK23 – + – + – + – +
RelA – – + + – – + +
Confined – – – – + + + +
i
A549
Rel. rate of confined migration
P = 0.0004
P = 5.27×10-5
NS
shCSK23 – + – +
RelA – – + +
j
H1299
Rel. rate of confined migration
P = 0.0047
P =0.0022
NS
shCSK23 – + – +
RelA – – + +
k
H460
Rel. rate of confined migration
P = 0.0008
P =0.002
NS
shCSK23 – + – +
RelA – – + +
l
A549
shCSK23 – + – +
Flag-ALDH1B1 – – + +
IB:ALDH1B1
IB:CSK23
IB:Actin
55
40
40
kDa
m
H1299
shCSK23 – + – +
Flag-ALDH1B1 – – + +
IB:ALDH1B1
IB:CSK23
IB:Actin
55
40
40
kDa
n
H460
shCSK23 – + – +
Flag-ALDH1B1 – – + +
IB:ALDH1B1
IB:CSK23
IB:Actin
55
40
40
kDa
o
H1299
Rel. lipid peroxidation
P = 0.006
P = 0.0015
P = 0.0006
NS
shCSK23 – + – + – + – +
ALDH1B1 – – + + – – + +
Confined – – – – + + + +
p
H1299
Cell death (%)
P = 0.0002
P = 0.001
P = 0.0002
NS
shCSK23 – + – + – + – +
ALDH1B1 – – + + – – + +
Confined – – – – + + + +
q
H460
Cell death (%)
P = 4.2×10-5
P = 0.0024
P = 0.0013
P=0.0437
shCSK23 – + – + – + – +
ALDH1B1 – – + + – – + +
Confined – – – – + + + +
r
H1299
Rel. rate of confined migration
P = 0.0002
P = 1.42×10-6
P = 0.0004
shCSK23 – + – +
ALDH1B1 – – + +
s
H460
Rel. rate of confined migration
P = 0.0156
P = 0.0002
NS
shCSK23 – + – +
ALDH1B1 – – + +
t
shCSK23 – +
EV
ALDH1B1
3039
41231
Brain luciferase intensity
P = 0.0051
P = 0.0137
NS
shCSK23 – + – +
ALDH1B1 – – + +

Supplementary Fig. 10 CSK23 is required for the survival and migration of confined cells by activating NF-kB and ALDH1B1 expression

a-c CSK23-depleted A549, H1299 or H460 cells overexpressing EV or RelA were analyzed by immunoblotting. The samples derive from the same experiment but different gels for RelA and another for CSK23, Actin were processed in parallel (**a**). The samples derive from the same experiment but different gels for RelA and another for CSK23, Actin were processed in parallel (**b**). The samples derive from the same experiment but different gels for RelA, Actin and another for CSK23 were processed in parallel (**c**). **d, e** CSK23-depleted A549 or H1299 cells overexpressing EV or RelA were assessed for lipid peroxidation by BODIPY 581/591 C11 staining. **f-k** CSK23-depleted A549, H1299 or H460 cells overexpressing with EV or RelA were stained with Annexin-V-PE after 6 hours of transwell migration. The percentages of unconfined and confined cell death were quantified. (**f-h**). Transwell migration assays were performed after 8 or 18 hours of culture and the numbers of migrated cells were quantified (**i-k**). **l-n** CSK23-depleted A549, H1299 or H460 cells overexpressing EV or ALDH1B1 were analyzed by immunoblotting. The samples derive from the same experiment but different gels for ALDH1B1 and another for CSK23, Actin were processed in parallel (**l**). The samples derive from the same experiment but different gels for ALDH1B1 and another for CSK23, Actin were processed in parallel (**m**). The samples derive from the same experiment but different gels for ALDH1B1, Actin and another for CSK23 were processed in parallel (**n**). **o** CSK23-depleted H1299 cells overexpressing EV or ALDH1B1 were stained with BODIPY 581/591 C11 to assess lipid peroxidation in unconfined and confined cells. **p-s** CSK23-depleted H1299 or H460 cells overexpressing EV or ALDH1B1 were stained with Annexin V-PE. The percentages of unconfined and confined cell death were quantified (**p**, **q**). Transwell migration assays were performed and the numbers of migrated cells were quantified (**r**, **s**). **t** Representative images of brain metastasis (Fig. 6**k**) from $n = 5$ mice per group (left). Data represent mean ± SD of luciferase intensities per mouse (right). Data are presented as mean ± SD ($n = 3$ biologically independent experiments) (**d-k, o-s**). Immunoblotting experiments were performed with the indicated antibodies. Data are representative of three independent experiments (**a-c, l-n**). *P*-values were calculated using unpaired two-tailed Student's *t* test (**d-k, o-t**). NS, not significant. Rel., relative. Source data are provided as a Source Data file.

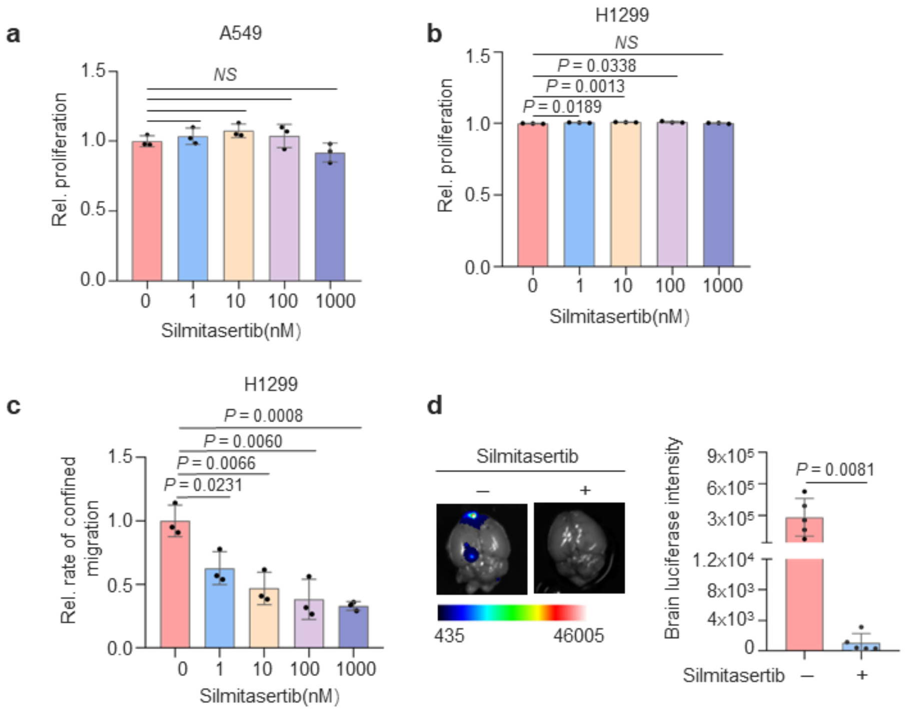

Supplementary Fig. 11 CSK23 inhibitor suppresses tumor cell migration and metastasis

a, b A549 or H1299 cells were treated with 0/1/10/100/1000 nM silmitasertib. Cell proliferation assay was performed for 24 hours. Relative cell proliferation of A549 and H1299 cells was normalized to 0 μM respectively. **c** H1299 cells were treated with 0/1/10/100/1000 nM silmitasertib. Transwell migration assays were performed and the numbers of migrated cells were quantified. **d** Representative images of brain metastasis (Fig. 6**n**) from *n* = 5 mice per group (left). Data represent mean ± SD of luciferase intensities per mouse (right). Data are presented as mean ± SD (*n* = 3 biologically independent experiments) (**a-c**). *P*-values were calculated using unpaired two-tailed Student's *t* test (**a-d**). NS, not significant. Rel., relative. Source data are provided as a Source Data file.
