# Supplementary material for: Compression-induced NF-κB activation sustains tumor cell survival in confinement by detoxifying aldehydes and promotes metastasis
Source: Nat Commun. 2025 Dec 14;17:778. doi: 10.1038/s41467-025-67452-7 (PMC12824153; doi:10.1038/s41467-025-67452-7)
Supplement: Supplementary file 2 — Description of Addtional Supplementary Files [file 41467_2025_67452_MOESM2_ESM.pdf]

Description of Additional Supplementary Files

Supplementary Data 1. The sgRNA sequences for all metabolic enzyme genes.

Supplementary Data 2. A CRISPR-based screen of 1,684 metabolic enzymes in a lung metastasis model (*n* = 3 independent experiments). *P*-value for each sgRNA was calculated using two-tailed Student's *t* test, whereas *P*-value for each gene was calculated using one-tailed Student's *t* test (negative selection).

Supplementary Data 3. Mass spectrometry analyses of IKKβ-associated proteins after compression (*n* = 2 independent experiments). *P*-values were calculated using two-tailed Student's *t* test.

Supplementary Data 4. The aldehyde MRM transitions.

Supplementary Movie 1-4 Tumor cells migration in confining spaces.

EGFP-A549 and EGFP-H1299 cells expressing shNT or shALDH1B1 were subjected to PDMS migration assays. Before the assay, the cells were stained with DAPI. These cells were then loaded into the channel inlet for real-time tracking. Dead cells in the PDMS device showed positive DAPI staining (blue). The movies are as follows: EGFP-A549 shNT (Supplementary Movie 1), EGFP-A549 shALDH1B1 (Supplementary Movie 2), EGFP-H1299 shNT (Supplementary Movie 3), and EGFP-H1299 shALDH1B1 (Supplementary Movie 4).
