# Supplementary material for: Compression-induced NF-κB activation sustains tumor cell survival in confinement by detoxifying aldehydes and promotes metastasis
Source: Nat Commun. 2025 Dec 14;17:778. doi: 10.1038/s41467-025-67452-7 (PMC12824153; doi:10.1038/s41467-025-67452-7)
Supplement: Supplementary file 8 — Reporting Summary [file 41467_2025_67452_MOESM8_ESM.pdf]

nature portfolio

Corresponding author(s): Weiwei Yang

Last updated by author(s): Nov 13,2025

Reporting Summary

Nature Portfolio wishes to improve the reproducibility of the work that we publish. This form provides structure for consistency and transparency in reporting. For further information on Nature Portfolio policies, see our Editorial Policies and the Editorial Policy Checklist.

Statistics

For all statistical analyses, confirm that the following items are present in the figure legend, table legend, main text, or Methods section.

n/a	Confirmed	
☐	☒	The exact sample size (*n*) for each experimental group/condition, given as a discrete number and unit of measurement
☐	☒	A statement on whether measurements were taken from distinct samples or whether the same sample was measured repeatedly
☐	☒	The statistical test(s) used AND whether they are one- or two-sided<br>*Only common tests should be described solely by name; describe more complex techniques in the Methods section.*
☒	☐	A description of all covariates tested
☒	☐	A description of any assumptions or corrections, such as tests of normality and adjustment for multiple comparisons
☐	☒	A full description of the statistical parameters including central tendency (e.g. means) or other basic estimates (e.g. regression coefficient) AND variation (e.g. standard deviation) or associated estimates of uncertainty (e.g. confidence intervals)
☒	☐	For null hypothesis testing, the test statistic (e.g. *F*, *t*, *r*) with confidence intervals, effect sizes, degrees of freedom and *P* value noted<br>*Give P values as exact values whenever suitable.*
☒	☐	For Bayesian analysis, information on the choice of priors and Markov chain Monte Carlo settings
☒	☐	For hierarchical and complex designs, identification of the appropriate level for tests and full reporting of outcomes
☐	☒	Estimates of effect sizes (e.g. Cohen's *d*, Pearson's *r*), indicating how they were calculated

Our web collection on statistics for biologists contains articles on many of the points above.

Software and code

Policy information about availability of computer code

Data collection	Immunofluorescence staining data: FV31S-SW (version 2.4.1)<br>Cell fluorescent dye staining data: Gen 5 (version 3.15), harmony (version 4.9)<br>Realtime PCR data: LightCycler 480 software (version 1.5.1)<br>Flow cytometry: CytoFlex 5 (Beckman Coulter), MA900 Multi-Application Cell Sorter (SONY Biotechnology)
Data analysis	Statistical analysis: GraphPad Prism (version 8.0)<br>CRISPR screening data analysis: MAGeCK algorithm (version 0.5.7), MAGeCKFlute R package (version 2.8.0).<br>GEO data analysis: R software (version 4.3.2)<br>Mass spectrometry analysis: MaxQuant (version 1.6.10.43)<br>Immunofluorescence staining analysis: Columbus (version 2.8.2)<br>Bioluminescence imaging analysis: IVIS-Image software (version 4.0)<br>Aldehyde analysis: Agilent MassHunter Quantitative Analysis (version 10.2)<br>Flow cytometry: CytExpert (version 2.4), MA900 cell sorter software (version 3.3.2)<br>Tumor area calculation: Image (version 1.49)

For manuscripts utilizing custom algorithms or software that are central to the research but not yet described in published literature, software must be made available to editors and reviewers. We strongly encourage code deposition in a community repository (e.g. GitHub). See the Nature Portfolio guidelines for submitting code & software for further information.

Data

Policy information about availability of data

All manuscripts must include a data availability statement. This statement should provide the following information, where applicable:

- Accession codes, unique identifiers, or web links for publicly available datasets
- A description of any restrictions on data availability
- For clinical datasets or third party data, please ensure that the statement adheres to our policy

Provide your data availability statement here.

Research involving human participants, their data, or biological material

Policy information about studies with human participants or human data. See also policy information about sex, gender (identity/presentation), and sexual orientation and race, ethnicity and racism.

Reporting on sex and gender	No sex and gender-based analysis was performed. The study focused on characterizing the pre-existing pathological phenomenon of tumor cells within confining vasculature, an objective cellular event that is not contingent on patient sex or gender.
Reporting on race, ethnicity, or other socially relevant groupings	No race, ethnicity or other socially relevant other socially relevant groupings were performed.
Population characteristics	One cohort of patients consisted of 61 lung cancer patients were included in the study, with 29 patients experiencing metastasis recurrence and 31 patients not showing metastasis recurrence. Immunofluorescence analyses of ALDH1B1 and RelA expression were conducted in all 61 lung cancer patients (Fig. 7a-7d). Immunofluorescence analyses of ALDH1B1 and 4-HNE expression were conducted in all 61 lung cancer patients (Fig. 7e-7g). Immunofluorescence analyses of ALDH1B1 and TUNEL staining were also performed in all 61 lung cancer patients (Fig. 7h and 7i). Immunofluorescence analyses of ALDH1B1 and 4-HNE expression were also performed in 29 lung cancer patients with metastasis recurrence and 31 lung cancer patients without metastasis recurrence (Fig. 7j and 7k).
Recruitment	Participants were not recruited.
Ethics oversight	The use of human lung tumor specimens and the database was approved by the Institutional Review Board at Shanghai Chest Hospital (KS(Y)23082) and Shanghai XinHua Hospital (XHEC-C-2025-155-1). Informed consent was obtained from all patients. . Informed consent was obtained from all patients.

Note that full information on the approval of the study protocol must also be provided in the manuscript.

Field-specific reporting

Please select the one below that is the best fit for your research. If you are not sure, read the appropriate sections before making your selection.

☒ Life sciences ☐ Behavioural & social sciences ☐ Ecological, evolutionary & environmental sciences

For a reference copy of the document with all sections, see nature.com/documents/nr-reporting-summary-flat.pdf

Life sciences study design

All studies must disclose on these points even when the disclosure is negative.

Sample size	The chosen sample size are based on the numbers used for previous publications (PMID: 31243371, PMID: 35315437), which is most optimal to generate statistically significant results. No statistical methods were used to predetermine sample sizes.
Data exclusions	No samples or animals were excluded from the analyses.
Replication	All replicates are biological replicates obtained from biologically independent experiments. All attempts at replication were successful. The sample size and number of replicates for each experiment is included in the figure legends.
Randomization	The samples/cells were randomized to be examined. The mice were randomized to put into separate groups/cages for experiments.
Blinding	For all experiments, the investigators were divided into two groups. One group was blinded to allocation during experiments and outcome assessment.

Reporting for specific materials, systems and methods

We require information from authors about some types of materials, experimental systems and methods used in many studies. Here, indicate whether each material, system or method listed is relevant to your study. If you are not sure if a list item applies to your research, read the appropriate section before selecting a response.

Materials & experimental systems

n/a	Involved in the study
☐	☒ Antibodies
☐	☒ Eukaryotic cell lines
☒	☐ Palaeontology and archaeology
☐	☒ Animals and other organisms
☒	☐ Clinical data
☒	☐ Dual use research of concern
☒	☐ Plants

Methods

n/a	Involved in the study
☒	☐ ChIP-seq
☐	☒ Flow cytometry
☒	☐ MRI-based neuroimaging

Antibodies

Antibodies used

Rabbit polyclonal anti-ALDH1B1 (115560-1-AP), rabbit monoclonal anti-IκBα (10268-1-AP) and Flag (20543-1-AP), and mouse monoclonal antibodies against Actin (60008-1-Ig) and RelA (66535-1-Ig) were purchased from Proteintech (Wuhan, China). Mouse monoclonal antibody against Tubulin (T9026) was purchased from Sigma (St. Louis, MO, USA). Mouse IgG antibody (sc-2025) was purchased from Santa Cruz Biotechnology (CA, USA). Rabbit polyclonal anti-CSK23 (PA5-98299) was purchased from Thermo Fisher Scientific. Rabbit monoclonal antibodies against HA (3724), RelA (8242), Lamin B1 (12586s), IKKβ (8943), IKKβ Ser177/181 (2697) and mouse monoclonal anti-IκBα Ser32/36 (9246) were purchased from Cell Signaling Technology. Goat polyclonal anti-CD31 (AF3628) was purchased from Novus Biologicals. Mouse monoclonal anti-4-HNE (MAB3249) was purchased from R&D Systems.
The following secondary antibodies were used: goat-anti-mouse IgG second antibody (31160, Thermo), goat-anti-rabbit IgG second antibody (31210, Thermo), donkey-anti-mouse Alexa Fluor 488 (A21202, Thermo), donkey-anti-rabbit Alexa Fluor cy3 (711-165-152, Jackson ImmunoResearch) and donkey-anti-goat Alexa Fluor 647 (A21447, Thermo). For immunoblotting analysis, the antibodies and their dilutions were as follows: ALDH1B1 (1:1000), CSK23 (1:1000), Flag (1:4000), HA (1:4000), Actin (1:4000), Tubulin (1:5000), RelA (1:2000), IKKβ (1:1000), IκBα pS32/36 (1:1000), IKKβ pS177/181 1:1000), IκBα (1:4000), Lamin B1 (1:4000). For immunofluorescence analysis, the antibodies and their dilutions were as follows: CD31(1:100), ALDH1B1(1:200), 4-HNE(1:200), RelA(1:200).

Validation

All commercial antibodies were validated by manufactures for indicated species and applications. Validation procedures are described in the websites of the manufactures:
Anti-ALDH1B1: Applications: WB, IHC, IF/ICC, IP, ELISA. Reactivity: human, mouse, rat.
Anti-IκBα: Applications: WB, IHC, IF, FC, IP, CoIP, ELISA. Reactivity: human, mouse, rat, pig, chicken, bovine, ducks.
Anti-Flag: Applications: WB, IHC, IF, IP, CoIP, ChIP, RIP, ELISA. Reactivity: all.
Anti-Actin: Applications: WB, IP, IHC, IF, FC, CoIP, ELISA. Reactivity: human, mouse, rat, plant, zebrafish.
Anti-RelA: Applications: WB, IHC, IF, CoIP, ChIP, RIP, ELISA. Reactivity: human, pig, chicken, bovine
Anti-Tubulin: Applications: WB, IF. Reactivity: human, mouse, rabbit, bovine, yeast, chicken, fungi, amphibian.
Mouse IgG: Applications: WB, IP, IHC. Reactivity: all.
Anti-CSK23: Applications: WB, IHC, ELISA, IP. Reactivity: human.
Anti-HA: Applications: WB, IP, IHC, IF, F, ChIP. Reactivity: all.
Anti-RelA: Applications: WB, IP, IHC, IF, F, ChIP. Reactivity: human, mouse,rat, hamster, monkey, dog.
Anti-Lamin B1: Applications: WB. Reactivity: human, mouse,rat.
Anti-IKKβ: Applications: WB, IP. Reactivity: human, mouse,rat, monkey.
Anti-IKKβ Ser177/181: Applications: WB, IHC, F. Reactivity: human, mouse,rat, hamster, monkey.
Anti-IκBα Ser32/36: Applications: WB. Reactivity: human, mouse,rat, monkey.
Anti-CD31: Applications: WB, F, IHC, F/ICC. Reactivity: human, mouse,rat.
Anti-4-HNE: Applications: WB, IHC, IF. Reactivity: all.
Goat-anti-mouse IgG second antibody: Applications: WB, IF, IHC, IP. Reactivity: mouse.
Goat-anti-rabbit IgG second antibody: Applications: WB, IF, IHC, IP. Reactivity: rabbit.

Eukaryotic cell lines

Policy information about cell lines and Sex and Gender in Research

Cell line source(s)

HEK293T cells and human lung cancer cell lines A549, H1299 and H460 were obtained from the Type Culture Collection of the Chinese Academy of Sciences.

Authentication

Cells were authenticated using the short tandem repeat (STR) method.

Mycoplasma contamination

All cell lines tested negative for mycoplasma contamination.

Commonly misidentified lines
(See ICLAC register)

No commonly misidentified cell lines were used in this study.

Animals and other research organisms

Policy information about studies involving animals; ARRIVE guidelines recommended for reporting animal research, and Sex and Gender in Research

Laboratory animals	BALB/C athymic nude mice (female, 6 weeks) were purchased from Lingchang Biotech (Shanghai, China).
Wild animals	The study did not involve wild animals.
Reporting on sex	All mice were female .Sex was not considered as a biological variable in the study design. This is because the primary objective was to investigate the intrinsic behavior of A549 tumor cells under confinement in vivo. The use of a single-sex cohort was chosen to minimize experimental variability and establish a clear baseline for assessing the effects on tumor cell survival and metastasis in this specific context.
Field-collected samples	No field-collected samples were used.
Ethics oversight	Animal experiments were approved by the Institutional Animal Care and Use Committee (IACUC) of Center for Excellence in Molecular Cell Science (CEMCS) and complied with all relevant ethical regulations (No. SIBCB-S355-2306-18).

Note that full information on the approval of the study protocol must also be provided in the manuscript.

Plants

Seed stocks	*Report on the source of all seed stocks or other plant material used. If applicable, state the seed stock centre and catalogue number. If plant specimens were collected from the field, describe the collection location, date and sampling procedures.*
Novel plant genotypes	*Describe the methods by which all novel plant genotypes were produced. This includes those generated by transgenic approaches, gene editing, chemical/radiation-based mutagenesis and hybridization. For transgenic lines, describe the transformation method, the number of independent lines analyzed and the generation upon which experiments were performed. For gene-edited lines, describe the editor used, the endogenous sequence targeted for editing, the targeting guide RNA sequence (if applicable) and how the editor was applied.*
Authentication	*Describe any authentication procedures for each seed stock used or novel genotype generated. Describe any experiments used to assess the effect of a mutation and, where applicable, how potential secondary effects (e.g. second site T-DNA insertions, mosiacism, off-target gene editing) were examined.*

Flow Cytometry

Plots

Confirm that:

☒ The axis labels state the marker and fluorochrome used (e.g. CD4-FITC).

☒ The axis scales are clearly visible. Include numbers along axes only for bottom left plot of group (a 'group' is an analysis of identical markers).

☒ All plots are contour plots with outliers or pseudocolor plots.

☒ A numerical value for number of cells or percentage (with statistics) is provided.

Methodology

Sample preparation	Cells with or without ALDH1B1 depletion were harvested and labeled with PE-conjugated Annexin V using the Annexin V-PE/7-AAD kit (BD Biosciences) following the manufacturer's protocol. The stained cells were then analyzed using flow cytometry (CytoFlex 5, Beckman Coulter). In in vivo CRISPR screening, twenty-four hours after tail vein injection, the mice's lungs were collected and digested. The cells were then harvested and stained with DAPI (1:5000). The viable EGFP-positive tumor cells were subsequently sorted using the MA900 (Sony Biotechnology).
Instrument	CytoFlex 5 (Beckman Coulter) and MA900 (Sony Biotechnology).
Software	CytExpert (version 2.4) and MA900 cell sorter software (version 3.3.2).
Cell population abundance	Cells were used when purity was >90%.
Gating strategy	Starting cells were gated by FSC/SSC gates. The boundary between "positive" and "negative" was defined by the single positive control and the negative control.

☒ Tick this box to confirm that a figure exemplifying the gating strategy is provided in the Supplementary Information.
